# Sedation and Analgesia for Intubation, LISA, and INSURE Procedures in Israeli NICUs: Caregivers’ Practices and Perspectives

**DOI:** 10.3390/jcm14165865

**Published:** 2025-08-19

**Authors:** Rasha Zoabi Safadi, Ayala Gover, Naama Tal Shahar, Irit Shoris, Arina Toropine, Adir Iofe, David Bader, Morya Shnaider, Arieh Riskin

**Affiliations:** 1 Rappaport Faculty of Medicine, Technion Israel Institute of Technology, Haifa 3525433, Israelariskin@technion.ac.il (A.R.); 2 Department of Neonatology, Bnai Zion Medical Center, Haifa 3339419, Israel

**Keywords:** premedication, NICU, Israel, intubation, LISA, INSURE

## Abstract

**Background/Objectives**: Early pain exposure in newborns is linked to negative short- and long-term outcomes. Preterm infants often require endotracheal intubation for mechanical ventilation or brief laryngoscopy for surfactant administration via Less Invasive Surfactant Administration (LISA) or Intubation–Surfactant–Extubation (INSURE). While premedication before intubation is well-studied, data regarding premedication for LISA/INSURE are limited. We aimed to explore premedication practices for intubation and LISA/INSURE procedures across Neonatal Intensive Care Units (NICUs) in Israel. **Methods:** An anonymous online questionnaire comprising 27 questions about premedication practices was distributed to neonatal caregivers in Israel. The questions addressed the use of premedication before intubation or LISA/INSURE, the existence of written protocols, pharmacological agents employed, and caregiver satisfaction with the medications used. **Results:** Questionnaires were collected between January and July 2023, yielding 69 responses from 20 NICUs. Almost all respondents (95.7%) routinely use premedication before intubation, but only 65.7% use it for LISA/INSURE. For non-emergency intubations, extremely low-birth-weight (ELBW) infants received premedication less often than the general neonatal population (75.4% vs. 95.7%, respectively). Most caregivers (91.2%) did not report increased procedure failure associated with premedication during LISA/INSURE. The vast majority of Israeli caregivers do not include muscle relaxants in their premedication regimen for intubation. Dual therapy regimens yielded higher satisfaction rates than monotherapy. Higher complication rates, particularly respiratory depression, were observed with Fentanyl, especially when used as monotherapy. **Conclusions:** Significant variations exist in premedication practices among caregivers across Israeli NICUs. Premedication is commonly administered for intubation but is considerably less frequent for LISA/INSURE, despite these procedures also being painful. ELBW infants received less premedication. Notably, muscle relaxants are infrequently used for premedication by Israeli NICU caregivers.

## 1. Introduction

Historically, it was believed that neonates, particularly preterm infants, were incapable of perceiving pain [1]. Consequently, this vulnerable population underwent painful procedures, including major surgeries, without analgesia [2]. Subsequent research, however, revealed that early and repeated painful stimuli can cause adverse neurological effects and negative long-term consequences for infants [3,4,5,6].

Premature infants in NICUs are frequently exposed to painful diagnostic and therapeutic interventions. A systematic review of observational studies indicated that neonates experience an average of 7.5 to 13.3 painful procedures per day during their NICU stay [7]. While infants now typically receive premedication and analgesia during surgery, protocols for premedication during other painful NICU procedures vary significantly between units [8,9].

Endotracheal intubation, although often life-saving, is a painful procedure. One common indication is surfactant administration in preterm infants with Respiratory Distress Syndrome (RDS). Alternative methods for surfactant delivery that still require laryngoscopy—a painful stimulus—include LISA and INSURE. Unlike intubation for mechanical ventilation, infants undergoing LISA/INSURE ideally maintain spontaneous breathing, which presents a challenge when selecting premedication agents [10,11]. In LISA, surfactant is administered via a thin catheter directly into the trachea under laryngoscopy [12], while the INSURE procedure involves intubation, surfactant administration through the endotracheal tube, and immediate extubation [13].

Published data on premedication practices for neonatal intubation worldwide indicate that the vast majority of elective intubations are performed with premedication [8,9,14,15,16,17,18]. However, other surveys have shown the inconsistent use of premedication or practices that do not adhere to current recommendations [16,17].

Variations in the specific drugs and combinations used for premedication before intubation have been highlighted globally, though fewer studies have specifically addressed premedication for LISA/INSURE [9,19,20,21,22,23,24]. Maintaining spontaneous respiration during LISA and INSURE is crucial, posing a challenge for drug selection, as opposed to premedication for intubation where apnea is less of a concern.

This study aimed to describe standard premedication practices in Israel for intubation and other painful procedures involving laryngoscopy, such as LISA and INSURE. Our survey explored the pharmacological agents most commonly used, allowing for a comparison to be made with international data and the assessment of the need for standardized national recommendations.

## 2. Materials and Methods

This cross-sectional study was conducted among NICUs across Israel. The survey was developed using Google Docs, and the link was distributed to caregivers via the national neonatal email network and the WhatsApp application. The questionnaire was anonymous and consisted of 27 questions directed at caregivers working in Israeli NICUs, including neonatologists, neonatology fellows, NICU nurse practitioners, and general physicians working in NICUs. The questionnaire was reviewed by a team of neonatologists and researchers to ensure clarity and relevance to the target audience of Israeli neonatal caregivers. It was then piloted with a group of 6 caregivers whose responses were collected and analyzed. Personal communication was established with at least one contact person in each participating department. Through this communication, representation of at least 20 of Israel’s 26 NICUs was confirmed. Responses were collected over seven months, from January 2023 to July 2023.

The questionnaire collected general demographic information regarding the responder’s position and the size of their NICU. Subsequent questions addressed the use of premedication before intubation and LISA/INSURE, the pharmacological agents used, the administration of Atropine, the administration of muscle relaxants, caregiver satisfaction with the medications, and their opinion on the need for uniform national guidelines for sedation and analgesia in these contexts.

Data were entered into Microsoft Excel (Microsoft Office, Seattle, WA, USA) for descriptive statistical analysis. Comparisons were made using the Chi-square test for categorical data and the Kruskal–Wallis ANOVA for the ranks or Mann–Whitney U test for non-parametric continuous data (SigmaPlot, version 11.0, Systat Software Inc., San Jose, CA, USA). Results are presented as percentages or medians.

## 3. Results

The survey was conducted from January to July 2023. Seventy responses were received; one was excluded due to incomplete data. The final analysis includes 69 responses representing at least 20 NICUs. Partial responses for specific questions were included in the analysis where data were available or were otherwise excluded from the calculation for that specific question.

- Responder Demographics:

Respondents included senior physicians (42%), neonatal nurse practitioners (24.6%), department heads (14.5%), unit heads (11.6%), neonatology fellows (4.3%), and NICU general physicians (2.9%).

Most respondents (63.8%) had over 10 years of experience in neonatology; 17.4% had 6–10 years; and 18.8% had 1–5 years.

The majority (62.3%) worked in medium-sized NICUs (3000–6000 births/year), and 27.5% worked in large centers (>6000 births/year).

- Pharmacologic Agents Used:

Fentanyl was the most commonly used agent (56.5%), either alone or in combination. Midazolam was the second most common.

Among the 54 responses detailing specific doses and combinations (78% of total responses), 19 caregivers (35% of the 54) reported using dual therapy, most frequently Fentanyl and Midazolam (48.3% of dual therapy users). However, the majority (35 caregivers, 65% of the 54) used monotherapy, with Fentanyl being the most common single agent (55% of monotherapy users).

Most caregivers (82.3%) never use muscle relaxants as part of premedication.

Atropine was administered prophylactically by 56.5% of caregivers, while 43.5% do not use it.

- Elective Intubation vs. LISA/INSURE:

The vast majority of respondents (95.7%) administer premedication before elective intubation, whereas only 63.8% do so prior to LISA or INSURE.

Most caregivers (91.2%) did not perceive an increase in LISA/INSURE procedure failure when using premedication.

**Figure 1:**
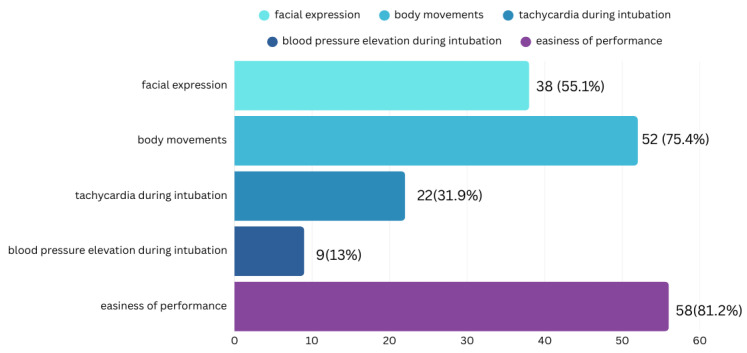
Caregiver assessment of premedication efficacy in preterm infants. This bar graph shows the methods caregivers use to assess the effect of premedication on preterm infants. The *y*-axis lists the assessment parameters, while the *x*-axis indicates the number of caregivers who use each one, with the corresponding percentage noted in parentheses. When rating the perceived premedication effect (scale 1 = no effect to 5 = maximal effect), 47.8% indicated a sufficient but not maximal effect (rating of 4). Among those reporting sufficient effects, dual therapy regimens received better ratings than monotherapy. The combination of Midazolam and Fentanyl had a high median rating with a substantial number of users (*n* = 14), while Propofol with Fentanyl had the highest median but fewer users (*n* = 5).

- Premedication Effectiveness and Procedure:

Caregivers typically assess the effectiveness of premedication. Most respondents evaluating premedication effectiveness used a combination of indicators, most commonly the ease of procedure performance and patient movement (see Figure 1).

**Figure 2:**
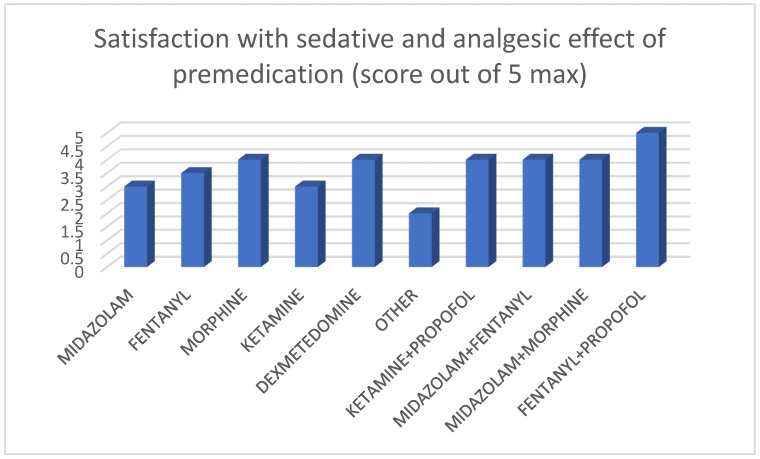
Caregiver satisfaction with sedative and analgesic effects of premedication. This bar graph presents the **median satisfaction scores** caregivers reported for the sedative and analgesic effects of different premedication drugs. The *X*-axis displays the specific medications administered, and the *Y*-axis represents the satisfaction score, ranging from 1 (**not satisfied at all**) to 5 (**very satisfied**). This figure provides a clear comparison of caregiver perceptions.

The differences in the median satisfaction ratings among treatment groups were statistically significant (*p* = 0.004) (Figure 2).

Most caregivers (68.2%) indicated needing fewer attempts to complete procedures when premedication was administered.

Regarding medication preparation time, 84% reported needing 5–10 min. Among these, Fentanyl was used by 33.7%, and the Fentanyl/Midazolam combination was used by 20%. Overall, Fentanyl was associated with the shortest preparation time.

When asked about the ease of procedure performance with premedication, caregivers most favored combinations of Fentanyl/Midazolam (38%) or Fentanyl/Propofol (30%).

- Premedication Complications:

Caregivers were asked about complications experienced following premedication in the past year. The most common was apnea (reported by 56.7%), followed by desaturation (43.3%), mild bradycardia (36.7%), and severe bradycardia requiring resuscitation (26.7%). Chest wall rigidity, a known serious complication particularly associated with Fentanyl and remifentanil, was reported by 5% of caregivers in the last year.

Among caregivers reporting respiratory depression, Fentanyl was the most frequently implicated drug (51%), predominantly when used as monotherapy (53%). Fentanyl was also the drug most commonly associated with the need for resuscitation.

- Standardized Protocols:

At least 77% of Israeli NICUs were represented. Of these, 76% reported having a written departmental protocol for premedication.

There was disagreement regarding the need for a uniform national sedation protocol for intubation/LISA/INSURE: 68.1% were in favor, while 31.9% were opposed.

Table 1 presents the results of a survey on premedication practices for various neonatal procedures. The first column lists the specific contexts, including elective and emergent intubation, as well as less invasive methods like LISA/INSURE. The subsequent columns show the total number of respondents and the number and percentage of caregivers who answered “Yes”, “No”, or “Sometimes” for each practice. The data provide a clear overview of the variation in premedication use across different procedures.

## 4. Discussion

This study aimed to describe premedication practices before intubation and LISA/INSURE procedures in Israel. We found that the vast majority of Israeli NICU caregivers routinely administer premedication before elective intubation. This rate is comparable to or slightly higher than the percentages reported in most previous worldwide surveys, although many of those reports were published some time ago, and practices may have evolved [8,14,15,16,17,18].

A notable discrepancy exists in attitudes towards premedication for LISA/INSURE compared to intubation. Although LISA/INSURE involves similarly painful stimuli (laryngoscopy and intratracheal surfactant instillation) [11,12,13], only 63.77% of surveyed caregivers routinely provide premedication for these procedures. While most department heads indicated agreement within their departments on premedicating for LISA/INSURE, about 24.4% reported ongoing debate among NICU physicians. This finding aligns with international studies [21,22,23]. A Spanish survey found that 77% of NICUs premedicated before LISA/INSURE [21], while a UK publication showed only 50% provided premedication for LISA compared to universal premedication for elective intubation [22].

The primary reason for this variation likely stems from the need to maintain spontaneous breathing during and after LISA/INSURE, as most sedative and analgesic agents can cause respiratory depression. Hypotension, another potential side effect of some agents, can also complicate patient management, especially in smaller, less stable neonates [24,25,26].

While studies have confirmed that premedication reduces pain and discomfort, no single drug or combination has proven definitively superior. In our survey, Fentanyl, alone or in combination, was the most common premedication agent (56.5%). This may reflect adherence to national neonatal pain guidelines, which include Fentanyl as an option for intubation analgesia [27]. This aligns with the global findings: opioids, sometimes with muscle relaxants, are recommended internationally [23]. A European survey found that opioids were the most common choice for LISA [28], and the UK NEOPRINT survey reported Fentanyl (60.3%) or morphine (23%) use for endotracheal intubations, with Fentanyl being the first-line choice for LISA in 66% of cases [22].

Although Fentanyl is widely accepted [23,29], alternative analgesics warrant consideration, especially for LISA/INSURE where opioid side effects, including the rare but catastrophic chest wall rigidity [30], could lead to procedure failure. Among these alternatives, Propofol has been the subject of several investigations. A randomized trial and an observational study on low-dose Propofol for LISA showed improved infant comfort (reduced ComfortNeo scores), though this was accompanied by an increased need for transient non-invasive ventilation [19,20]. Mild and transient side effects for propofol were shown in another trial [31]. A different study directly compared ketamine with Propofol for premedication before LISA and found both drugs to be similarly efficient and well-tolerated [32]. A third study specifically on ketamine found that it resulted in low pain scores while maintaining hemodynamic stability [33]. These findings suggest that Propofol and ketamine may be effective alternatives, and we currently await the results of the multicenter PROLISA study [34], which is designed to further investigate Propofol premedication before LISA. Dexmedetomidine was found effective for LISA premedication without significant side effects in one study [35]. This body of research highlights the ongoing need to explore and optimize alternative premedication strategies.

Due to known neonatal vagal hyper-reactivity, prophylactic Atropine administration is sometimes employed. Our study found that about half of caregivers (56.5%) use Atropine prophylactically. However, robust evidence supporting its routine inclusion is lacking. A previous randomized trial showed less heart rate decrease in groups receiving Atropine (alone or with pancuronium) compared to no premedication [36].

Anesthesiologists often recommend combination therapy to achieve optimal hypnosis, analgesia, and muscle relaxation, recognizing drug interactions [37,38]. While dual therapy is common in Pediatric Intensive Care Units (PICUs) [39,40], practice varies in NICUs, with monotherapy being the most studied. In our study, caregivers rated dual therapy (particularly Fentanyl/Midazolam) as providing better sedation effectiveness, as perceived by the responders, compared to monotherapy, a statistically significant difference (*p* = 0.004).

Interestingly, Israeli neonatologists rarely use muscle relaxants as premedication before elective intubation (reported by only 12%). This likely reflects concerns about eliminating spontaneous breathing, which poses challenges (requiring effective mask ventilation) if intubation fails. However, based on the anesthesiology and neonatology literature, muscle relaxants should likely be considered for intubation premedication [23,40]. Randomized controlled trials have demonstrated the benefits of premedication for preterm infants. A combination of succinylcholine, morphine, and Atropine led to faster intubation with less bradycardia and less trauma than awake intubation. Similarly, infants who received pancuronium and Atropine experienced less hypoxia and a smaller increase in intracranial pressure compared to those who received no premedication or Atropine alone [36,41,42].

Complications of premedication before procedures involving laryngoscopy remain a concern, especially with LISA premedication. Respiratory depression (including apnea) was the most common complication reported (56.7%), most frequently associated with Fentanyl, particularly when used as monotherapy. Since respiratory depression is a known opioid side effect [30], Fentanyl’s high usage frequency in this cohort explains this finding. Analyzing dose-dependent effects was challenging due to sample size and response variability.

Our findings suggest that the use of premedication for extremely low-birth-weight (ELBW) infants is a source of practice variation. We observed that 75.4% of caregivers provided premedication for ELBW infants before intubation, a rate significantly lower than the 95.7% reported for the general neonatal population. This disparity aligns with previous research indicating that caregivers often consider patient weight when selecting premedication, showing a significantly lower use of premedication for very-low-birth-weight (VLBW) infants compared to larger infants [43]. This difference in practice likely reflects valid concerns about the pharmacological effects of sedatives and analgesics in this particularly fragile population. Despite these concerns, our findings highlight a potential gap in care.

Based on our findings and available evidence from the literature, we propose several recommendations for neonatal premedication practices:

Universal Premedication: Premedication should be considered a standard of care for all painful procedures involving laryngoscopy, including elective intubation, LISA, and INSURE, to mitigate pain and stress and improve long-term neurodevelopmental outcomes.

Optimal Drug Choice: Fentanyl remains the most commonly used and accepted agent for premedication, aligning with both Israeli guidelines and reports from the international literature. However, given the complication risks, especially with LISA/INSURE, other regimens such as Propofol and ketamine should be considered and investigated.

Prophylactic Atropine: The use of Atropine should be strongly considered for all invasive procedures involving laryngoscopy to reduce the risk of vagal-induced bradycardia, a known physiological response in neonates.

Muscle Relaxants: Caregivers should reconsider the infrequent use of muscle relaxants for elective intubation. Evidence suggests that their use can improve procedural success rates and reduce physiological stress, such as hypoxia and intracranial pressure changes. While concerns about failed intubation are valid, the benefits may outweigh the risks in controlled settings.

Written departmental protocols can promote consistent premedication practices, ensuring appropriate drug selection and dosing, potentially improving efficiency and safety. While 76% reported having departmental protocols, there was disagreement regarding the need for national guidelines, though the majority (68%) favored their development.

A key limitation of this study is its cross-sectional survey design, which makes it susceptible to recall bias and social desirability bias. Furthermore, the absence of a universally accepted definition for terms such as “procedural failure”, “complications”, and “sedation effectiveness” introduces potential for variability in caregiver interpretation, limiting the comparability and generalizability of the findings. Our survey-based methodology, which relied on caregiver recall, also did not allow us to collect precise, objective data on key procedural metrics such as the exact duration of intubation attempts or the need for redosing. Anonymity, while encouraging honest responses, made it difficult to precisely track participation across all NICUs, though representation of the majority (at least 20/26) was confirmed. The relatively small sample size and wide variation in practices complicated the analysis and interpretation.

Despite these limitations, this is the first survey in Israel describing neonatal premedication practices for intubation and LISA/INSURE.

## 5. Conclusions

Among Israeli NICUs, great variations regarding premedication regimens were found.

In our study, there was a tendency for caregivers to use less premedication for LISA/INSURE compared to intubation. We found that ELBW infants may be receiving less premedication than the general premature infant population in Israeli NICUs. It was also observed that Israeli NICU caregivers infrequently use muscle relaxants before elective intubation—a practice that, based on the current literature, may warrant reconsideration. The strong support for uniform national guidelines among Israeli caregivers (68.1%) underscores a perceived need for standardization, likely reflecting the observed practice variations and a desire to improve patient outcomes and safety. This finding suggests a fertile ground for future policy initiatives and collaborative efforts to develop evidence-based national recommendations.

Further research and potentially standardized guidelines could help optimize neonatal premedication practices in Israel.

## Figures and Tables

**Table 1:** Caregiver responses regarding premedication practices for neonatal intubation, LISA, and INSURE.

	Number of Responders	Yes	No	Sometimes
Give premedication before elective intubation.	69	66 (95.7%)	3 (4.3%)	
Give premedication before emergent intubation.	69	4 (6%)	43 (62.3%)	22 (31.7%)
Give premedication before LISA/INSURE.	67	44 (65.7%)	23 (34.3%)	
Give premedication before elective intubation in ELBW.	69	52 (75.4%)	17 (24.6%)	
Use muscle relaxants as a part of premedication before intubation.	68	1 (1.5%)	56 (82.3%)	11 (16.2%)
Use atropine as part of premedication.	69	30 (43.5%)	39 (56.5%)	
Department heads indicate having written premedication protocols.	27	20 (74.1%)	7 (25.9%)	
Caregivers think that there is a need for uniform premedication protocols.	69	47 (68.1%)	22 (31.9%)	

## Data Availability

The raw datasets generated and analyzed during the current study are not publicly available due to participant privacy and confidentiality concerns.

## Abbreviations

The following abbreviations are used in this manuscript:

NICU	Neonatal Intensive Care Unit
LISA	Less Invasive Surfactant Administration
INSURE	Intubation–Surfactant–Extubation
ELBW	Extremely Low-Birth-Weight (preterm infants weighing ≤ 1000 g at birth)
VLBW	Very-Low-Birth-Weight (infants weighting ≤ 1500 g at birth)
RDS	Respiratory Distress Syndrome
